# The Effect of Pressure–Shift Freezing versus Air Freezing and Liquid Immersion on the Quality of Frozen Fish during Storage

**DOI:** 10.3390/foods11131842

**Published:** 2022-06-22

**Authors:** Ting Li, Shiyao Kuang, Ting Xiao, Lihui Hu, Pengcheng Nie, Hosahalli S. Ramaswamy, Yong Yu

**Affiliations:** 1 College of Biosystems Engineering and Food Science, Zhejiang University, 866 Yuhangtang Road, Hangzhou 310058, China; 15968336462@163.com (T.L.); 22113007@zju.edu.cn (S.K.); tinyaxiao980625@163.com (T.X.); xytsmf474@163.com (L.H.); pcn@zju.edu.cn (P.N.); 2 Key Laboratory of Equipment and Informatization in Environment Controlled Agriculture, Ministry of Agriculture, 866 Yuhangtang Road, Hangzhou 310058, China; 3 Department of Food Science, McGill University, 21111 Lakeshore Road, St-Anne-de-Bellevue, QC H9X 3V9, Canada; hosahalli.ramaswamy@mcgill.ca

**Keywords:** high pressure freezing, specialty freezing techniques, largemouth bass, freezing preservation, frozen food quality

## Abstract

In this study, a self-cooling laboratory system was used for pressure–shift freezing (PSF), and the effects of pressure–shift freezing (PSF) at 150 MPa on the quality of largemouth bass (Micropterus salmoides) during frozen storage at −30 °C were evaluated and compared with those of conventional air freezing (CAF) and liquid immersion freezing (LIF). The evaluated thawing loss and cooking loss of PSF were significantly lower than those of CAF and LIF during the whole frozen storage period. The thawing loss, L* value, b* value and TBARS of the frozen fish increased during the storage. After 28 days storage, the TBARS values of LIF and CAF were 0.54 and 0.65, respectively, significantly higher (*p* < 0.05) than the 0.25 observed for PSF. The pH of the samples showed a decreasing trend at first but then increased during the storage, and the CAF had the fastest increasing trend. Based on Raman spectra, the secondary structure of the protein in the PSF-treated samples was considered more stable. The α-helix content of the protein in the unfrozen sample was 59.3 ± 7.22, which decreased after 28 days of frozen storage for PSF, LIF and CAF to 48.5 ± 3.43, 39.1 ± 2.35 and 33.4 ± 4.21, respectively. The results showed that the quality of largemouth bass treated with PSF was better than LIT and CAF during the frozen storage.

## 1. Introduction

Fish deteriorates rapidly at ambient temperatures and, due to its high protein content, generally spoils faster than other muscle foods. The deterioration of fish is mainly due to the chemical and biochemical loss of quality and the subsequent deterioration caused by bacterial activity [1]. The deterioration of fish is a complex process involving physical, chemical and microbial mechanisms [2]. Therefore, freezing is a common storage method for fish. Freezing can greatly extend the shelf life of aquatic products, but with the extended storage, the quality of fish gradually declines. Several studies have shown that the moisture content and its distribution in meat tissues may change during the traditional freezing process [3]. Common commercial freezing methods can cause dense nucleation and excessive intracellular ice crystal growth, which can lead to the mechanical cracking of frozen foods [4,5]. As a new technology, high-pressure processing (HPP) at subzero temperatures has been shown to have great research potential due to frozen food phase transformations [6]. pressure–shift freezing (PSF) has been proven to be suitable for food freezing [7,8].

Pressure–shift freezing enables food to reach a very high degree of supercooling, and almost instantaneous (super-fast) decompression, and therefore the resulting freezing or crystallization process is extremely rapid [9,10]. pressure–shift freezing results in numerous small and evenly distributed ice crystal nuclei [11,12]. Therefore, PSF can greatly reduce or even eliminate the damage to tissue caused by freezing and effectively improve the quality of frozen products. It is considered an excellent alternative to traditional air and liquid immersion freezing techniques, but there is no commercial application case yet. Several studies have been carried out on the application of PSF for different foods. Zhu, Le Bail and Ramaswamy [5] compared the effects of different freezing methods on Atlantic salmon and found that high-pressure freezing produced large amounts of fine and regular intracellular ice crystals that were homogeneously distributed throughout the salmon. Su, Ramaswamy, Zhu, Yu, Hu and Xu [9] compared the sizes of ice crystals produced by water in muscle (shrimp and porcine liver) under pressure–shift freezing, conventional air freezing and liquid immersion freezing and showed that ice crystals produced by pressure–shift freezing were the smallest, most uniform and most evenly distributed in food. Similar conclusions were obtained in subsequent studies on abalone [13]. High pressure and freezing also have lethal effects on the microorganisms in foods [14,15,16], which is one of the advantages of pressure–shift freezing food.

However, PSF is affected by factors such as equipment size, pressurization rate, cooling method and temperature control, so that PSF research is limited to only some specialized laboratories with small research equipment that cannot be used at commercial levels. For example, in several studies, the pressure chamber designed is too small to provide effective temperature control, which makes it difficult to scale up for production [10,17]. On the other hand, obtaining a higher freezing point depression requires ethanol or silicone oil as a pressure transfer medium [9,13,18], and this increases the cost of PSF and may not be environmentally friendly. In previous studies at Zhejiang University [6,9], researchers used a simple self-cooling device that cools by the melting heat absorption of the cooling solution. This approach overcomes the limitations of previous studies that relied on more complex and expensive high-pressure equipment with jacketed cooling sources for pressure chamber cooling. Using this system, pressure–shift freezing can be applied to large-scale frozen food. The previous studies [6,9] also confirmed that fish PSF was practical and reduced thawing and cooking losses.

This study is focused on the quality of frozen stored fish (up to 28 days at −30 °C) following pressure–shift freezing (PSF) compared with conventional air freezing (CAF) and liquid immersion freezing (LIF). The pH, thiobarbituric acid reactive substances (TBARS), color, thawing and cooking losses, and protein secondary structure were used as quality parameters. The overall purpose was to provide a reference point for the high-quality freezing and storage of aquatic products.

## 2. Materials and Methods

### 2.1. Chemicals

Absolute ethyl alcohol was obtained from Hangzhou Hongda Chemical & Instrument Co., Ltd., Hangzhou, China. Trichloroacetic acid was obtained from Shanghai Aladdin Bio-Chem Technology Co., Ltd., Shanghai, China. Ethylene diamine tetraacetic acid was bought from Hangzhou Jiachen Chemical Co., Ltd., Hangzhou, China. 2-Thiobarbituric acid was bought from Shanghai Jinsui Bio-Technology Co., Ltd., Shanghai, China and 1,1,3,3-Tetraethoxy-propane (TEP) was bought from Beijing Belo Biotechnology Co., Ltd., Beijing, China. All chemicals used in this study were of reagent grade.

### 2.2. Sample Preparation

Twenty live largemouth bass were bought from a market near Zhejiang University and shipped refrigerated to the lab within half an hour. The fish were cut into pieces of about 10 g with an approximate size of 15 mm × 15 mm × 20 mm. A total of 180 pieces were randomly selected, placed in polyethylene bags, and stored at 4 °C until frozen by different methods and then frozen within 24 h using different freezing methods. Test samples were divided into 3 groups with 60 samples for each freezing group, which were frozen with either PSF, LIF or CAF. On days 1, 7, 14, 21 and 28, samples were taken from each group, three for thawing loss and cooking loss, three for color measurement and pH testing and three for TBARS measurement, and three were subjected to Raman analysis. The samples were directly placed in a conventional low-temperature freezer (−30 °C) for CAF freezing. LIF freezing was accomplished by immersing samples in a pre-chilled ethanol/water (50%, *v*/*v*) solution maintained in a conventional refrigerator at −30 °C.

Pressure–shift freezing was carried out as detailed in earlier studies [6,19] with minor modifications. A laboratory-scale high-pressure device was used for the HP treatments (Baotou Kefa High Pressure Technology Co., Ltd., Baotou, China) with a maximum chamber capacity of 5 L; pure water was used as the pressure transmission medium. The cooling system consisted of a polyvinyl chloride bottle with caps used as cooling medium containers; a chamber; the cooling medium (ice) and the sample container (nylon vacuum bag). Then, 300 mL cooling medium was filled in to the container, and a 50 mL centrifuge tube was attached to the cap and screwed into the bottle to squeeze out the excess water. The cooling medium container was frozen at −30 °C for 24 h, and then the centrifuge tube was pulled out to form a chamber with a diameter of 30 mm and a depth of 100 mm. The chamber ensured that the prepared sample container was placed at the center of the cooling system and reduced the freezing time of the sample. After the cooling system was completed, the test sample was introduced, and the system was placed in the pressure chamber for pressure–shift freezing. The chamber was pressurized to 150 MPa, held at the pressure for 240 s and then quickly depressurized. After releasing the pressure, the sample was immediately removed from the high-pressure chamber and quickly transferred to a pre-cooled (−30 °C) ethanol/water bath (50%, *v*/*v*) to complete the freezing process. After 1, 7, 14, 21 and 28 days of frozen storage, the samples were taken out for further analyses. Each group was thawed at 4 ± 0.5 °C for 24 h before measuring the indicators. Fresh largemouth bass served as a control group.

### 2.3. Determination of Thawing Loss and Cooking Loss

Thawing loss was determined according to the method of Xia, Kong, Liu and Liu [20] with some modifications. After freezing, the weight of each piece of fish (*W*_0_) was determined, and then the fish was placed in a refrigerator for controlled slow thawing to the central temperature of 4 °C, after which the sample was taken out of the polyethylene bag, surface dried with paper towel and weighed immediately (*W*_1_). The thawing loss was calculated with following equation:

(1) $$\mathrm{Thawing}\;\mathrm{loss}\;(\%)=\frac{W_{0}-W_{1}}{W_{0}}\times 100$$

The cooking loss was determined using the procedure of Faridnia, Ma, Bremer, Burritt, Hamid and Oey [21] with some modifications. The thawed sample was weighed (*W*_1_), and then the fish was placed in a food bag and heated in a water bath at 90 °C for 25 min. The cooked fish was allowed to cool at room temperature for 30 min. Then, the cooked sample was taken out of the food bag, immediately blot dried and weighed (*W*_2_). Cooking loss is calculated as:

(2) $$\mathrm{Cooking}\;\mathrm{loss}\;(\%)=\frac{W_{1}-W_{2}}{W_{1}}\times 100$$

### 2.4. Color Measurement

The color of each sample from the three fish pieces was measured using a spectrophotometer (CM-600D, Konica Minolta Sensing Inc., Tokyo, Japan). The color reader is first calibrated with the white board and then blank calibrated. L*, a* and b* were translated as indicators of lightness, redness and yellowness, respectively.

### 2.5. pH Value

Three pieces of fish meat were taken from each group of samples and cut into minced meat. Then, 5 g minced meat and 50 mL deionized water were taken in a beaker and stirred using a homogenizer (FJ200-SH, Shanghai Specimen and Model Factory, Shanghai, China) for 2 min. The homogenate was then centrifuged at 5000 rpm for 10 min at room temperature. The pH of the obtained supernatant was measured with a pH meter (FE-20, Mettler TolEDO Instruments Ltd., Shanghai, China).

### 2.6. Thiobarbituric Acid Reactive Substances (TBARS)

Thiobarbituric acid reactive substances (TBARS) was used to monitor the development of lipid oxidation in the largemouth bass samples during frozen storage. TBARS was determined using the method described by Zhang, Li, Lu, Shen and Luo [22], with modifications. First, 5 g of homogeneous sample was placed in a 100 mL conical flask with a stopper to which 50 mL of a 7.5% (*w*/*v*) solution of trichloroacetic acid was added and incubated in a thermostatic oscillator bath (HZ-9211KB, Taicang Hualida Experimental Instrument Co., Ltd., Suzhou, China) at 50 °C for 30 min with agitation (160 rpm). Then this was filtered with a double-layer quantitative slow-speed filter paper, the initial filtrate was discarded and the subsequent filtrate was used. Next, 5 mL of filtrate and 5 mL of 0.02 mol/L thiobarbituric acid (TBA) aqueous solution were added into a 10 mL test tube, and then stoppered. Then, the test tube was placed in a 90 °C thermostatic water bath (N3–8, Hangzhou Bioer Technology Co., Ltd., Hangzhou, China) for 30 min, taken out, and cooled to room temperature. The absorbance was measured at 532 nm with a microplate reader (SpectraMax190, Molecular Devices (Shanghai) Co., Ltd., Shanghai, China). TBARS (expressed as mg MDA/kg meat) was calculated by multiplying the absorbance readings by a factor of 10.2, which was obtained from a standard line prepared using 1,1,3,3-tetraethoxy-propane as a precursor of malonaldehyde.

### 2.7. Raman Spectroscopy Analysis

The Raman spectra were measured as described by Chen, Liu, Li, Wei and Li [23] with some modifications. Samples about 1 mm thick were placed on a Raman spectrometer (Labram HR Evolution, HORIBA Jobin Yvon, Paris, France). The sample was placed on the microscope and laser focused with a focal length lens, after which the spectrum was tested and collected. The parameters were set as follows: 50 times the objective lens; cycle 2 times; laser wavelength, 632.8 nm; spectral scan range, 400–2000 cm^−1^. Each sample was measured three times. The scanned spectra were smoothed, baseline corrected, normalized and averaged, and the Origin 2018 software was used to analyze the protein secondary structure content.

### 2.8. Statistical Analysis

All treatments were carried out in triplicate for each experimental condition, and all data were expressed as means ± standard deviation (SD). The significant differences between the results were analyzed by one-way analysis of variance (ANOVA). Duncan’s test (*p* < 0.05) was used to compare the differences in average values by using SPSS software (23.0, IBM Corp., Armonk, NY, USA).

## 3. Results and Discussion

### 3.1. Thawing and Cooking Loss

**Figure 1:**
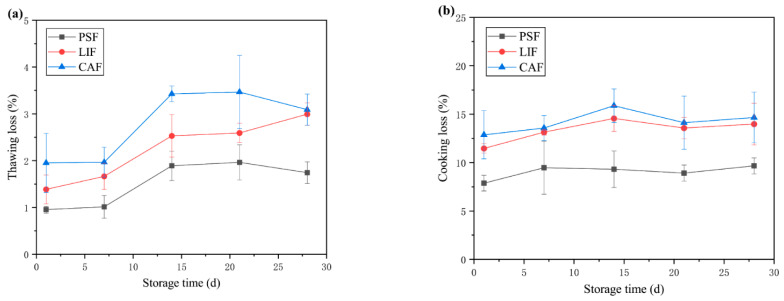
Thawing loss (**a**) and cooking loss (**b**) changes in largemouth bass during frozen storage with different freezing methods. Acronyms: PSF = pressure–shift freezing; LIF = Liquid immersion freezing; CAF = Conventional air freezing.

Drip loss is one of the major quality factors in frozen thawed food that has a bearing on both the freezing conditions (rapid vs. slow) and storage history (storage temperature, temperature fluctuations). Drip losses can be estimated from the product immediately on thawing or after subsequent cooking. The thawing and cooking losses are important indicators of the quality of frozen food, as they affect not only the economics of the food but also the weight loss and shrinkage during cooking, as well as the juiciness and tenderness of the meat [24,25]. Figure 1a shows the thawing loss for the largemouth bass samples during frozen storage. The results show that the thawing loss increased with the extension of storage time. Especially on day 14, there was a significant increase (*p* < 0.05) in thawing loss, although no significant change (*p* > 0.05) after that. The PSF thawing loss during the entire frozen storage process was significantly smaller (*p* < 0.05) than that of the LIF and CAF samples. The change in thawing loss could be related to ice crystal size [26]. PSF is expected to form small and uniform ice crystals in the sample, thereby maintaining the integrity of cells [9,10,12]. LIF and CAF could have resulted in slow freezing and therefore led to the formation of large ice crystals in the sample, disrupting the integrity of cells and muscle tissue and resulting in significant water loss during thawing. Tironi et al. [27] showed that PSF is an effective method for reducing the juice loss of sea bass after thawing. Chen et al. [23] showed similar results, with increased thawing loss during storage in sea bass with different freezing treatments.

The cooking losses in the largemouth bass chunks during frozen storage are shown in Figure 1b. There was no significant change (*p* > 0.05) in cooking loss for largemouth bass with prolonged frozen storage time. This is different from some previous studies that show that cooking loss increased with storage time [23,28]. There are a number of possible reasons: (1) The storage time in this study was not very long, maximum one month; (2) the freezer used in the studies might have had better freezing capacity of the freezer, and the better temperature stability might have reduced the recrystallization of ice crystals during storage; (3) for this species, the main reason for the increased cooking loss was perhaps the size of the ice crystals formed rather than the recrystallization process, and thus, during storage, the cooking loss did not show an expected increase. However, there were significant differences (*p* < 0.05) in the cooking loss of largemouth bass under the three different freezing methods. After 28 days of frozen storage, the cooking losses of PSF, LIF and CAF were 9.7%, 14.0% and 14.7%, respectively. Cooking loss also results from the damage to muscle structure caused by the heat-induced denaturation of the myofibrillar protein [26]. Another study [29] also reports that the cooking loss of fish samples treated with different freezing conditions can be explained by ice crystal size, muscle tissue destruction and protein denaturation.

### 3.2. pH Value

**Figure 2:**
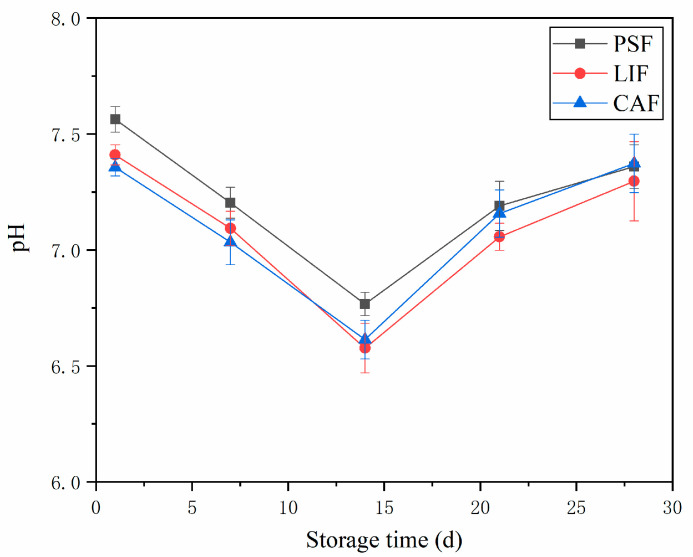
Changes in the pH of largemouth bass during frozen storage with different freezing methods. Acronyms: PSF = pressure–shift freezing; LIF = Liquid immersion freezing; CAF = Conventional air freezing.

As shown in Figure 2, the pH of largemouth bass with three different freezing treatments first decreased and then increased as a function of storage time. On the first day of frozen storage, the pH values of PSF, LIF and CAF were 7.56 ± 0.06, 7.41 ± 0.04 and 7.36 ± 0.04, respectively, and the pH of PSF was significantly higher (*p* < 0.05) than the values for LIF and CAF. The pH of PSF was also slightly higher than those for LIF and CAF during the early frozen storage. In previous studies, pressurization increased the pH of meat [13,30,31], and the reason for this pH increase may be that pressure-induced structural unfolding of muscle protein [31]. The pH of all samples decreased slightly up to the 14th day, which as reported in [32] could be caused by the bacterial fermentation of carbohydrates, resulting in the formation and accumulation of organic acids in fish meat. During storage, the glycogen substances that sustain life activities in the muscle of largemouth bass are degraded to form lactic acid, so the initial pH decreases. After 14 days, the in pH increases, possibly due to the activity of the endogenous enzymes present in fish, which can result in protein deamination and decarboxylation reactions and produce alkaline substances such as ammonia or amines, resulting in an increase in the pH [33]. Lin et al. [34] observed the lowest pH in frozen beef samples on the 8th day. In another study on sea bass [35], the pH showed the same change trend, decreasing first and then increasing. The lowest pH values appear at different storage times, which may be due to different species, muscle types and treatment methods. In the rising stage, the rate of pH increase associated with PSF was the least and that of CAF is the highest. This could be because PSF forms small and uniform ice crystals during the freezing process, while CAF is a slow freezing process with a low nucleation rate and only a few nuclei; it in turn forms larger and mostly extracellular ice crystals [5,9]. CAF caused extensive mechanical damage to cells, caused the outflow of substances in cells and accelerated the oxidation of proteins.

### 3.3. Color

Color is considered an important indicator for consumers to judge the quality of meat, and it influences consumers’ psychology and purchasing intent [36]. Table 1 shows the changes in the color parameters L*, a*, b* of the samples during frozen storage. L*, a* and b* of PSF at 150 MPa were no different from those from LIF and CAF (*p* > 0.05) at any point along the entire duration of storage. According to published results, pressure over 150 MPa during PSF is likely to cause significant (*p* < 0.01) color changes in pork muscle [37]; pressure higher than 325 MPa will affect the color in meat, while moderate pressure (up to 130 MPa) can improve the meat color and increase the redness [38].

As shown in Table 1, the lightness (L* value) increased with storage time, indicating that the color of the fish meat is getting somewhat brighter or could be paler from colored samples. The increase in the L* value may be due to the exudation of water from the fish meat during the thawing process, caused by ice crystals, and the formation of a water film on the surface of the fish meat that can enhance the reflection or refraction of light. Lin et al. [34] treated beef with different methods, found that the L* values of the samples increased first during storage and credited it to changes in the refractive index of the meat surface caused by drip exuding. On the other hand, the redness (a* value) of the samples did not change significantly during frozen storage. However, other studies with pork [39] have reported a decrease in a* value due to the myoglobin denaturation and the formation of metmyoglobin during the frozen storage. The a* value of beef was reported to show a trend of an initial increase and a subsequent decrease [34]. In a different study on beef [40], myoglobin that came into contact with oxygen in the air converted to oxymyoglobin, and then with the deepening of oxidation, metmyoglobin gradually replaced oxymyoglobin, and the color of meat changed from bright red to dark brown. The differences in a* values may also be due to the different sample types. Largemouth bass meat is relatively white in color and has higher fat content but lower myoglobin content than pork and beef. Changes in yellowness (b* value) were similar to those in L* value, both increasing with storage time. This could be due to the oxidation of fish fat during freezing storage. This result is similar to that of Pinheiro et al. [41], who studied the frozen storage of lamb at −25 °C and reported that lipid oxidation leads to increased L* and b* values.

### 3.4. Thiobarbituric Acid Reactive Substances

**Figure 3:**
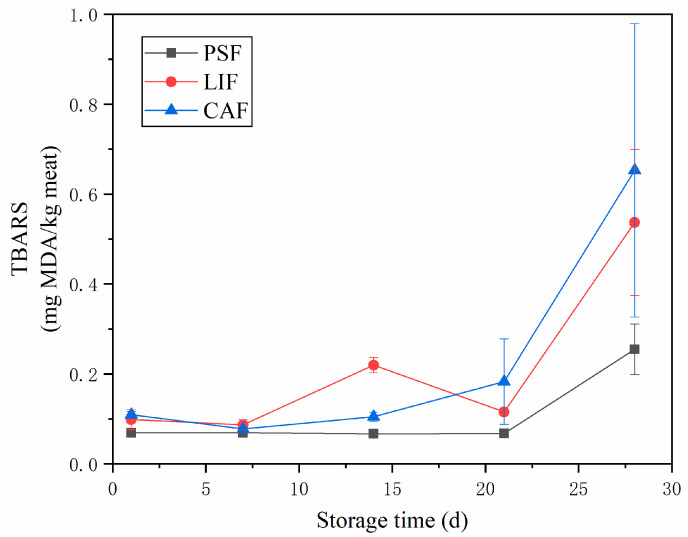
TBARS changes for largemouth bass during frozen storage with different freezing methods. Acronyms: PSF = pressure–shift freezing; LIF = Liquid immersion freezing; CAF = Conventional air freezing.

Lipid oxidation not only affects the flavor, texture and taste of food but also reduces the nutritional value of food [42]. Thiobarbituric acid reactive substances (TBARS, also called TBA-i) are usually used to evaluate lipid oxidation in meat products. The larger the TBARS, the more severe the lipid oxidation. The changes in TBARS in largemouth bass during 28 days of frozen storage are shown in Figure 3. As expected, for all three freezing treatments, the TBARS increased gradually with the prolongation of storage time. Further, on the final (i.e., 28th) day of storage, the TBARS of LIF and CAF were 0.54 and 0.65 mg MDA/kg meat, respectively, significantly higher (*p* < 0.05) than the 0.25 mg MDA/kg meat of PSF. We cannot explain the reason for the sharp rise in the TBARS of LIF on day 14, which could have been because of experimental uncertainties. An increase in TBARS has been associated with the decomposition of hydroperoxides into secondary oxidation products, especially aldehydes [43]. With the extension of storage time, the gap between the TBARS of PSF with LIF and CAF became larger. Benjakul and Bauer [44] suggested that the increase in TBARS may be due to the increase in solute concentration caused by freezing and the destruction of muscle fiber structure by ice crystals, releasing pro-oxidants such as phospholipase, lipase and heme iron in cells into muscle and leading to muscle fat oxidation. The slow conventional freezing (CAF and LIF) from the surface to center of samples can form a progressively growing ice crest, causing tissue rupture and uneven crystal structure as well as pressure build-up on the residual unfrozen fraction in the samples; this in turn causes biological tissue damage and cell rupture [45] and results in more pro-oxidants release. PSF utilizes a high degree of super-cooling under the high-pressure hold, followed by rapid depressurization, resulting in uniform and very small ice crystals throughout the sample [5]. Therefore, PSF can greatly reduce or avoid conventional freezing problems such as tissue deformation and cell shrinkage [9,46]. This explains why under the same storage condition, the TBARS of CAF and LIF were higher than that of PSF. Lin et al. [38] found similar of TBARS of slow-frozen beef that was higher than that of static magnetic field extended supercooling (SM-ES) treated beef under similar frozen storage conditions. After 6 months of frozen storage, the TBARS of samples frozen at −8/−18 °C were reported to increase by 444% [39].

### 3.5. Protein Secondary Structure

**Figure 4:**
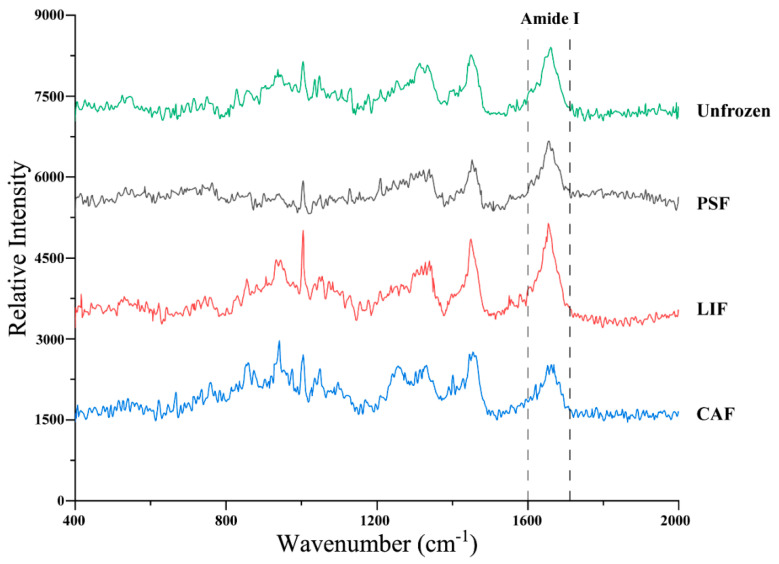
Raman spectra of largemouth bass proteins from unfrozen fish and fish frozen under different freezing methods stored for 28 days in the 400–2000 cm^−1^ region. Acronyms: PSF = pressure–shift freezing; LIF = Liquid immersion freezing; CAF = Conventional air freezing.

Raman spectra are very sensitive to molecular vibrations and are rarely affected by water solvents [23]. Therefore, this method is widely used to study the secondary structure of proteins and peptides. The Raman spectra (400–2000 cm^−1^) of 28-day frozen stored and unfrozen samples are shown in Figure 4. The amide bonds of proteins have several different vibrational modes, of which the band at 1600–1700 cm^−1^ is the most studied. This range is linked to the stretching vibration of C–N and C=O bonds, associated with the protein bone structure, and is ofteit n used to evaluate the secondary structure of proteins [47,48]. In general, a high proportion of α-helix protein structure in the amide I band mainly appears in the 1650–1660 cm^−1^ band, while β-sheet and β-turn appear at 1680 cm^−1^ and 1665–1680 cm^−1^ bands, respectively. The amide I band centered at 1660–1665 cm^−1^ has been attributed to random coils or disordered structures within the secondary protein structure [49,50]. The frequency and intensity changes in the Raman bands have been used as main indicators of changes in the secondary protein structures and of variations in the local environments of myofibrillar proteins [51].

During protein denaturation, α-helix are converted to β-sheet and β-turn, and then β-sheets are converted to the random coil. Therefore, the content of α-helix and β-sheet can be used as indicators of protein stability, where higher α-helix and β-sheet content indicate more stable secondary structure [48]. The changes in the secondary structure content of the different largemouth bass amide I proteins are shown in Table 2. The content of α-helix in protein after frozen storage was significantly lower (*p* < 0.05) than that of the unfrozen samples. Meanwhile, the α-helix content in the protein of PSF samples was significantly higher (*p* < 0.05) than that of the CAF and LIF samples, showing that frozen storage leads to helix expansion. The stability of the α-helix structure is related to the hydrogen bonding between the carbonyl oxygen (−CO) and the amino hydrogen (NH−) in the polypeptide chain [52]. The oxidative cleavage of the hydrogen bond between the C=O and NH-groups results in reduced α-helix content [53]. The CAF samples exhibited the lowest α-helix content and the highest random coil content, indicating that their secondary structure was relatively unstable. The α-helix content decreased and the random coils content increased in the myofibrillar protein of sea bass during frozen storage, and the increase in random coils content came at the expense of α-helix [23]. PSF showed good protection of protein structure. This may be because the PSF effectively created numerous tiny ice crystals that remained small during the finish freezing and frozen storage, caused reduced cell damage, and improved the stability of the protein structure of largemouth bass after freezing.

## 4. Conclusions

This study evaluated the frozen storage of largemouth bass by pressure–shift freezing and compared it with conventional air freezing and liquid immersion freezing. Our study sought to identify a novel freezing method with minimal impact on the quality of seafood. The study demonstrated that PSF was the best freezing method to maintain high quality of frozen largemouth bass during storage. Both the thawing loss and the cooking loss of the PSF samples during frozen storage were significantly lower (*p* < 0.05) than with CAF and LIF. Thawing loss increased with storage time for all three freezing methods, but cooking losses were not different (*p* > 0.05). In this study, the pressure selected for PSF treatment was 150 MPa, and the PSF meat colors were not significantly different (*p* > 0.05) from those with CAF and LIF after the storage. At the same time, based on the changes associated with pH and TBARS, the quality of frozen bass was found to be affected by the storage time, due to lipid oxidation. Raman spectra showed the secondary structure of protein in PSF-treated samples to be more stable. Moreover, the PSF in this study adopts a new self-cooling system, developed in our laboratory, that has good potential adaptability for commercial high-pressure processing equipment and possible industrial applications. Using this system, other than the cost of high-pressure equipment, the operating cost for pressure–shift freezing is low, and the feasibility is high from a commercial point of view. In conclusion, compared with other conventional freezing techniques, PSF can better maintain the quality of largemouth bass during frozen storage and can be used as a promising freezing method in the food industry.

## Figures and Tables

**Table 1:** The effects of different freezing treatments on the color of largemouth bass during frozen storage.

Treatments	Time (Days)	L*	a*	b*
PSF	1	38.8 ± 2.19 ^a^	−3.61±0.33 ^a^	−1.35 ± 0.69 ^a^
	7	38.5 ± 0.91 ^a^	−3.38 ± 0.12 ^a^	−0.74 ± 0.56 ^a^
	14	40.2 ± 2.81 ^a,b^	−3.80 ± 0.46 ^a^	−0.07 ± 0.74 ^a,b^
	21	42.1 ± 2.66 ^a,b^	−3.47 ± 0.52 ^a^	0.39 ± 0.25 ^a,b^
	28	43.8 ± 3.33 ^b^	−3.71 ± 0.14 ^a^	1.15 ± 1.66 ^b^
LIF	1	36.3 ± 2.45 ^a^	−2.95 ± 0.49 ^a^	−1.66 ± 0.27 ^a^
	7	38.8 ± 1.75 ^a,b^	−3.32 ± 0.09 ^a^	−1.48 ± 0.53 ^a^
	14	38.9 ± 0.80 ^a,b^	−3.15 ± 0.27 ^a^	−0.50 ± 0.99 ^a,b^
	21	40.8 ± 1.00 ^b^	−3.23 ± 0.02 ^a^	0.54 ± 0.69 ^b^
	28	44.2 ± 0.61 ^c^	−3.39 ± 0.23 ^a^	1.13 ± 1.63 ^b^
CAF	1	37.0 ± 0.58 ^a^	−3.13 ± 0.50 ^a^	−0.89 ± 0.14 ^a^
	7	39.59 ± 0.26 ^b^	−3.46 ± 0.27 ^a^	−0.49 ± 0.53 ^a^
	14	39.4 ± 0.48 ^b^	−3.44 ± 0.18 ^a^	−0.97 ± 0.20 ^a^
	21	41.2 ± 1.16 ^c^	−3.21 ± 0.37 ^a^	−0.49 ± 0.79 ^a^
	28	43.8 ± 1.20 ^d^	−3.52 ± 0.19 ^a^	0.43 ± 1.39 ^a^

The results were expressed as the mean ± standard deviation (*n* = 3), and the letters (^a–d^) in the same column indicate significant differences (*p* < 0.05) between the data of different samples. Note: PSF, pressure–shift freezing; LIF, Liquid immersion freezing; CAF, Conventional air freezing.

**Table 2:** Secondary amide I protein structure contents in different largemouth bass samples (unfrozen and in frozen storage under different freezing methods for 28 days).

Secondary Structure Content (%)	α-Helix	β-Sheet	β-Turn	Random Coil
Unfrozen	59.3 ± 7.22 ^a^	23.7 ± 5.20 ^a^	6.14 ± 2.61 ^a^	10.8 ± 2.68 ^a^
PSF	48.5 ± 3.43 ^b^	26.5 ± 6.78 ^a^	11.2 ± 3.47 ^a^	13.8 ± 3.36 ^a^
LIF	39.1 ± 2.35 ^c^	29.9 ± 3.71 ^a^	11.4 ± 3.06 ^a^	19.7 ± 3.19 ^b^
CAF	33.4 ± 4.21 ^c^	24.2 ± 3.76 ^a^	20.3 ± 3.41 ^b^	22.1 ± 3.16 ^b^

The results were expressed as the mean ± standard deviation (*n* = 3), and the letters (^a–c^) in the same column indicate significant differences (*p* < 0.05) between the data in the different samples. Note: PSF, pressure–shift freezing; LIF, Liquid immersion freezing; CAF, Conventional air freezing.

## Data Availability

Data is contained within the article.
